# A structural perspective of how T cell receptors recognize the CD1 family of lipid antigen–presenting molecules

**DOI:** 10.1016/j.jbc.2024.107511

**Published:** 2024-06-28

**Authors:** Thinh-Phat Cao, Adam Shahine, Liam R. Cox, Gurdyal S. Besra, D. Branch Moody, Jamie Rossjohn

**Affiliations:** 1 Infection and Immunity Program and Department of Biochemistry and Molecular Biology, Biomedicine Discovery Institute, Monash University, Victoria, Australia; 2 School of Chemistry, University of Birmingham, Birmingham, United Kingdom; 3 Institute of Microbiology and Infection, School of Biosciences, University of Birmingham, Birmingham, UK; 4 Division of Rheumatology, Inflammation and Immunity, Brigham and Women’s Hospital, Harvard Medical School, Boston, Massachusetts, USA; 5 Institute of Infection and Immunity, Cardiff University, School of Medicine, Cardiff, UK

**Keywords:** CD1, lipid, T cell recognition, structure, lipid analog, antigen presentation

## Abstract

The CD1 family of antigen-presenting molecules adopt a major histocompatibility complex class I (MHC-I) fold. Whereas MHC molecules present peptides, the CD1 family has evolved to bind self- and foreign-lipids. The CD1 family of antigen-presenting molecules comprises four members—CD1a, CD1b, CD1c, and CD1d—that differ in their architecture around the lipid-binding cleft, thereby enabling diverse lipids to be accommodated. These CD1–lipid complexes are recognized by T cell receptors (TCRs) expressed on T cells, either through dual recognition of CD1 and lipid or in a new model whereby the TCR directly contacts CD1, thereby triggering an immune response. Chemical syntheses of lipid antigens, and analogs thereof, have been crucial in understanding the underlying specificity of T cell–mediated lipid immunity. This review will focus on our current understanding of how TCRs interact with CD1–lipid complexes, highlighting how it can be fundamentally different from TCR-MHC-peptide corecognition.

Studies on T cell–mediated immunity have centered on peptide antigen (Ag) display by major histocompatibility complex (MHC) proteins. Here, MHC-restricted αβ T cells, *via* their αβ T cell receptor (TCR), play a fundamental role in protective immunity toward pathogens. Conversely, T cells are also associated with aberrant immune responses when recognizing self-peptides presented by MHC molecules, leading to immune dysfunction and autoimmune diseases. However, despite the focus on MHC-mediated immunity over the last 40 years, there is another Ag-presenting system, termed CD1, comprising four transmembrane anchored Ag-presenting molecules, (CD1a, CD1b, CD1c, and CD1d), along with the soluble CD1e lipid transfer protein, that display Ags to T cells. Namely, CD1 molecules bind and display lipid-based Ags to T cells. These lipid-reactive T cells are also emerging as key players in protective immunity and immune dysfunction. Whereas nearly all our understanding of T cell Ag recognition that triggers immune responses is centered on peptides, the discovery of the CD1 family provides a new perspective that cellular, synthetic, and microbial lipids are often overlooked, but potentially relevant Ags for T cells. Moreover, it is now coming to light that lipids can promote inflammation in a CD1-dependent manner and cause aberrant immune responses. Because lipids, one of the four basic biological macromolecules, are structurally stable and cannot undergo point mutations to escape detection like peptides, the lipid Ag-presenting CD1 molecules are nonpolymorphic among individuals. In terms of foreign lipids derived from infectious agents, the lipid structures are unique, as further described in the subsequent part of this review. Thus, it is important to understand the fundamentals underpinning TCR recognition of CD1-presenting lipid Ags, which are universally applicable. Here, we highlight the important role that organic chemistry has played in unravelling the specificity underpinning TCR recognition of lipid Ags.

### Structure of CD1 molecules at a glance

CD1a, CD1b, CD1c, and CD1d adopt a three-dimensional fold similar to that of MHC class I molecules. However, unlike MHC molecules, whose Ag-binding clefts are ideally suited to capture peptides, CD1 molecules bind lipids (Fig. 1). Structural studies have shown that the shape of the CD1 Ag-binding clefts is different, thereby implying altered modes of lipid Ag display (1). Moreover, each isoform has a specific cell distribution profile and cellular trafficking pathway. For example CD1a, CD1c, and CD1d are expressed on nonoverlapping subsets of dendritic cells, and each human CD1 protein differs in expression of endosomal recycling motifs in their cytoplasmic tails (2).

**Figure 1:**
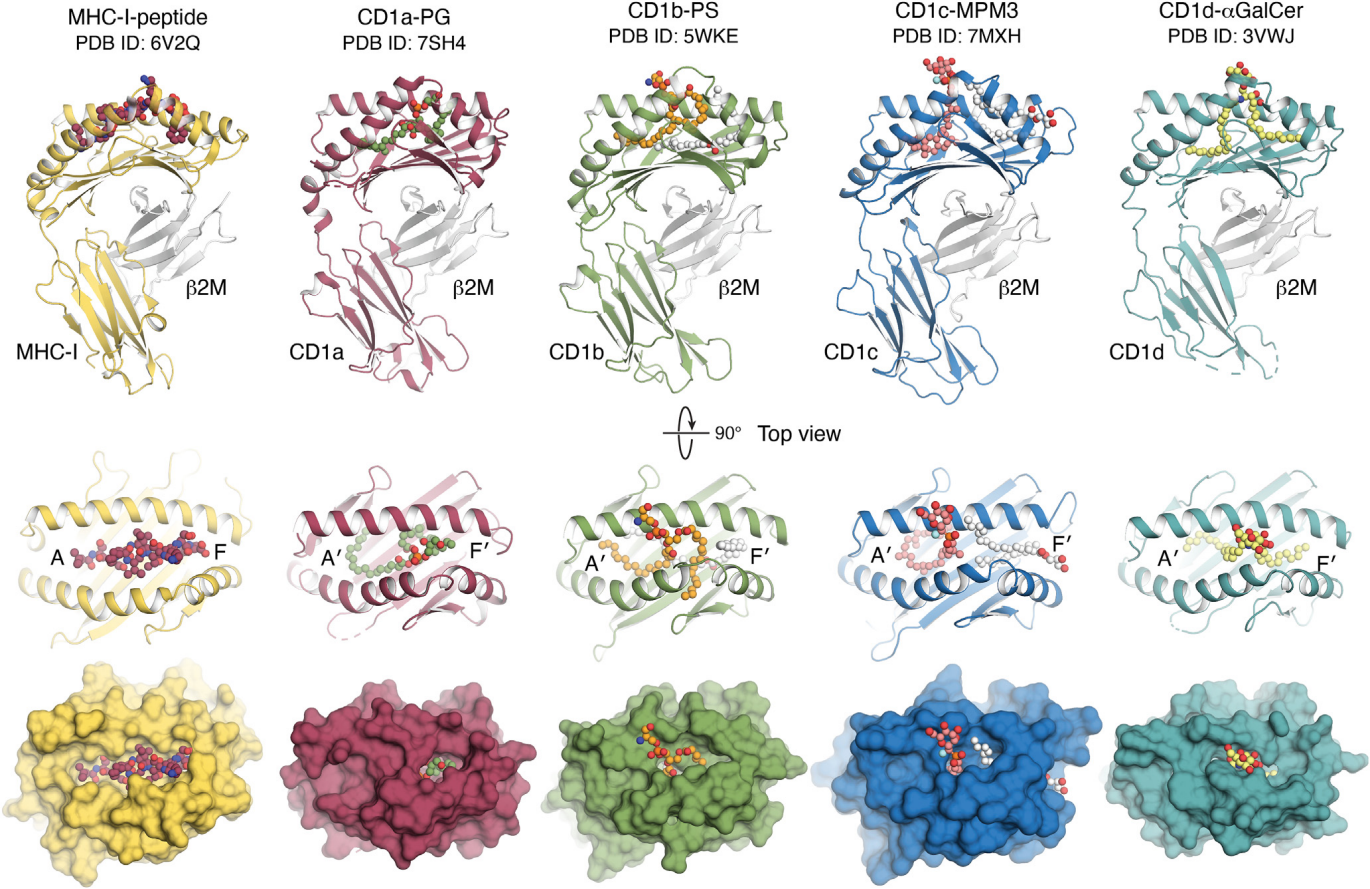
**CD1 at a glance.***Top*, structures of four CD1 isoforms in comparison with an MHC-I molecule (HLA-B∗57:03). Despite sharing similarity of overall β2M-associated fold, MHC-I has a shallow cleft for peptide binding, whereas CD1 molecules possess hydrophobic pockets to accommodate lipids. *Middle*, *top view* to the lipid binding pockets of CD1 molecules in comparison with the peptide binding cleft of MHC-I. The pockets A′ and F′ in CD1 molecules, corresponding to A and F in MHC-I, are indicated. *Down*, surface representation demonstrates the full exposure of the peptide in MHC-I, whereas only the lipid headgroups can protrude out of the binding pockets in CD1 molecules. αGalCer, α-galactosyl ceramide; MHC-I, major histocompatibility complex class I; MPM3, difluoromethylene-mannoside phosphomycoketide; PG, phosphatidylglycerol; PS, phosphatidylserine.

The lipids presented by the CD1 system differ in size and composition of their hydrophilic head groups (Fig. 2), and each human CD1 isoform has a cleft that likewise differs in size and architecture. Thus, the repertoires of lipids bound by each of the four CD1 isoforms may either overlap or be isoform specific. Examples of pan-CD1 ligands that bind multiple human CD1 isoforms (3) include self-lipids, such as phospholipids, sphingolipids, acylglycerols, and fatty acids (1). However, due to the different capacity and characteristics of their binding clefts, even lipid classes that show detectable binding to all four CD1 isoforms can still show marked preferences for specific isoforms. For example, sphingolipids are more efficiently captured by CD1a and CD1d, where CD1a shows a strong preference for molecules with long overall chain length C42 (or greater) (4), whereas efficient sphingomyelin (SM) capture by CD1d does not require long chain length. In terms of exogenous lipids, the presented lipids appear more structurally specific for individual CD1 proteins. Extensive studies described the complex and unique mycobacterial lipids that are presented by each isoform, including dideoxymycobactin by CD1a (5), diacylated sulfoglycolipid (6), mycolic acid (7) and glucose monomycolate (8) by CD1b, and phosphomycoketide (PM) and mannosyl-β1-phosphomycoketide (MPM) by CD1c (9). Additionally, CD1a and CD1c can present several classes of hydrophobic compounds that are small in size and therefore buried inside the CD1 protein, so that TCRs directly contact CD1 itself; these include contact sensitizers, such as farnesol, geranylgeraniol, urushiol, benzyl cinnamate, benzyl benzoate, and monoacylglycerol (10, 11). Another class of nonlipidic small molecule including phenyl pentamethyldihydrobenzosulfonate and its derivatives is presented by CD1d (12, 13) to Crohn's disease–associated invariant T cells (14, 15). Collectively, the structural, biochemical, and cellular studies, including CD1 tetramer and mass spectrometry approaches, have been critical to discover the factors within the lipid Ags that drive TCR recognition and downstream T cell responses.

**Figure 2:**
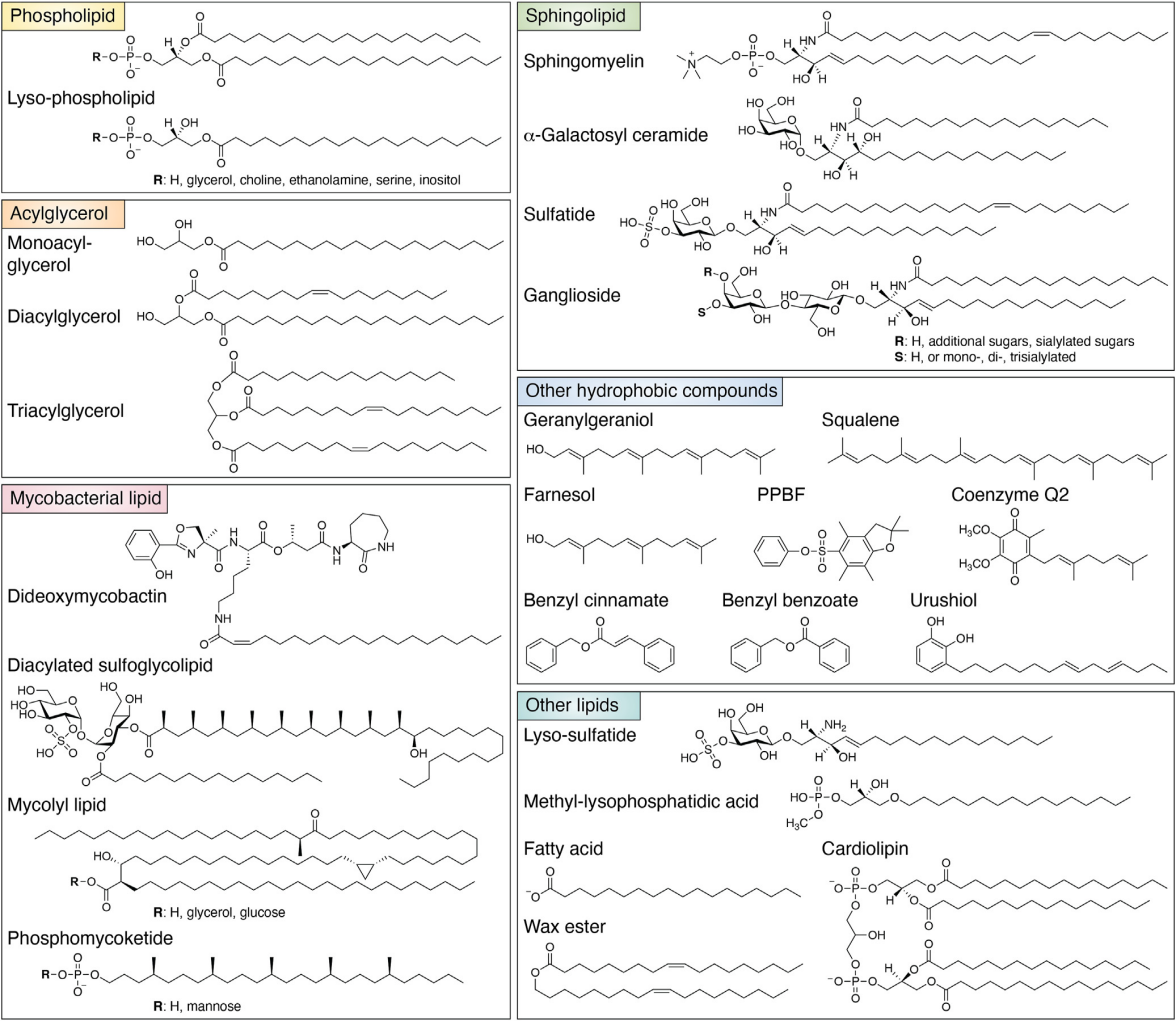
**Classes of lipids and hydrophobic compounds presented by CD1.** Chemical schematic of different classes of ligands that can bind to CD1.

### Insights into TCR recognition of CD1 molecules

TCR-peptide-MHC recognition has been extensively studied, and is defined by a central paradigm of “corecognition” (16), wherein a TCR epitope is constituted by both the peptide Ag and the polymorphic binding cleft of the MHC molecules. A TCR is a heterodimer of either α and β or γ and δ chains, which in turn defines the subpopulation to which the expressing T cell belongs. αβ and γδTCRs are indeed identical in their overall structural organisation (Fig. 3), except for the highly variable CDR regions, namely CDR3, which constitute the paratope. The same paradigm of corecognition can be observed in the TCR–CD1–lipid axis but is not universal. As the hydrophobic tails are buried inside the CD1 cleft, the exposed hydrophilic headgroup generally defines the TCR epitope. Presently, two major binding mechanisms are supported by solved ternary crystal structures: “corecognition” which occurs for all CD1 isoforms, and “absence of interference” for CD1a and CD1c, where the lipid is not directly contacted and its role is to permit CD1-TCR contact.

**Figure 3:**
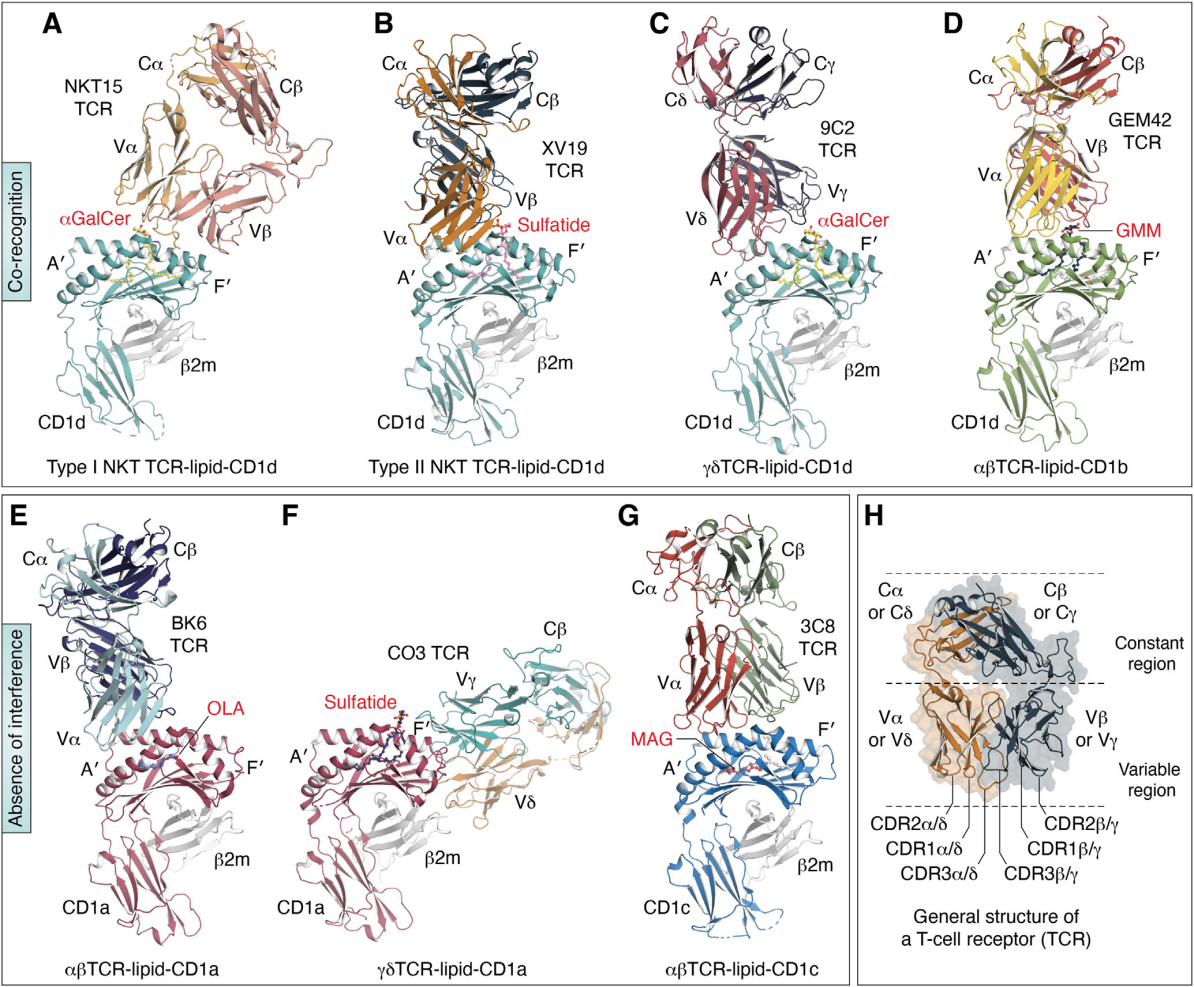
**Overall docking topologies of TCR-CD1-lipid.** Two paradigms of TCR–lipid–CD1 interactions. *A*, type I NKT TCR (NKT15) to CD1d-αGalCer (3VWJ (68)). *B*, type II NKT TCR (XV19) to CD1d-sulfatide (4EI5 (69)). *C*, 9C2 γδTCR to CD1-αGalCer (4LHU (28)). *D*, GEM42 αβTCR to CD1b-GMM (5L2K (53)). *E*, BK6 αβTCR to CD1a-oleic acid (4X6D (64)). *F*, CO3 γδTCR to CD1a-sulfatide (7RYN (66)). *G*, 3C8 αβTCR to CD1c-MAG (6C09 (11)). *H*, general structure of a T cell receptor (TCR). αGalCer, α-galactosylceramide; MAG, monoacylglycerol; GMM, glucose monomycolate; NKT, natural killer T.

### NKT TCR recognition of CD1d-lipid

Within the CD1 system, CD1d-lipid recognition by natural killer T (NKT) cells has been the most studied molecular paradigm. Two subsets of NKT cells are found, namely: type I NKT cells express invariant TCR α-chain (human TRAV10/TRAJ18, murine TRAV11/TRAJ18) paired with a limited set of β-chains (human TRBV25-1, murine TRBV1, TRBV13-2, or TRBV29), where α-galactosylceramide (αGalCer) (17) is the prototypical ligand, though other ligands can be recognized (18, 19, 20, 21, 22, 23, 24, 25). Type II NKT cells express a diverse repertoire of TCRs and do not recognize αGalCer, with the most widely studied ligand being sulfatide. CD1d-lipid recognition by the NKT TCRs can adopt distinct interaction modes for each subset: tilted and parallel docking mode over the F′ pocket of CD1d for type I NKT TCR *versus* orthogonal positioning above the A′ pocket for type II NKT TCR (Fig. 3, *A* and *B*). In the type I NKT TCR interaction, the αGalCer headgroup is solely contacted by the invariant TCR α-chain, while the TCR β-chain contacts the F′ pocket of CD1d. In the type II NKT TCR interaction, the TCR α-chain primarily contacts CD1d, while the TCR β-chain interacts with the sulfatide headgroup. However, one type II NKT TCR, namely A11B8.2, interacts with CD1d presenting a mycobacterial lipid adopting a parallel docking mode that resembles type I NKT TCR docking (26), thereby indicating that diverse type II NKT TCR usage can lead to diverse footprints atop CD1d.

Further, a minor subset of NKT cells has been identified as “atypical,” which includes clones whose characteristics fit neither type I nor type II NKT cells. This subset of atypical NKT cells may simultaneously recognize αGalCer but show diverse range of TCR gene usage. The interaction topology of one such atypical TCR resembles type II NKT TCR-CD1d-sulfatide complex, which mainly binds the A′ roof of CD1d (27). Indeed, in a number of TCR-CD1d structures, the A′ roof of CD1d appears to be the preferable docking platform of the TCRs, including γδTCR recognition (28) (Fig. 3*C*). TCR contact with the A′ roof is notable because MHC proteins lack a large contact surface that is not occupied by Ag, but the large lipid Ag-free surface of the A′ roofs seen in all four human CD1 proteins and mouse CD1d, serves as a landing pad for CD1-autoreactive TCRs (29).

#### Synthetic αGalCer analogs

Within these NKT TCR–CD1d–lipid interactions that define corecognition, the surface area of contact with the lipid is limited when compared to CD1d itself, yet the interaction between TCR and lipid is critical to direct the downstream functional responses. For example, different versions of αGalCer that activate type I NKT cells with varying potency can lead either to Th1 or Th2 effector responses that promote or inhibit the overall outcome of the immune response (30). Th1 and Th2 subsets are two types of CD4^+^ T cells that distinguish themselves by two typical patterns of cytokine productions [reviewed in (31)]. Thus, the key purpose is to design analogues that can bias desired pattern toward the desired immune response. Over the past decade, several groups have synthesized 100s of structurally diverse NKT cell agonists, some of which induce distinct and potentially therapeutically useful immune responses. However, a lack of effective delivery vectors and a tendency for some of the most potent and promising NKT cell activators to induce NKT cell anergy and depletion post stimulation has hindered their development for clinical application. As a result highly efficient synthetic protocols now allow us to access a diverse range of small-molecule NKT cell activators (see Fig. 4 for examples), including: (i) glycosyl ceramides (incorporating, *e.g.*, amide isosteres, different acyl chains), which represent structural variants of the proto-typical CD1d agonist αGalCer (32, 33, 34, 35, 36, 37, 38, 39, 40, 41, 42, 43, 44); (ii) αGalCer dimers used to probe multivalency effects (41); and (iii) nonglycosidic analogs based around the truncated αGalCer analog, threitol ceramide, which show increased bioavailability (45, 46). Many of these molecules are potent NKT cell activators; some are Th1, Th2, or Th17 cytokine-biasing, and show significant promise as immunomodulatory agents for a variety of therapeutic applications [reviewed in (47, 48, 49)]. In addition, the adaptability of the synthetic methodologies have also allowed the late-stage incorporation of a range of molecules, including haptens and labels (biotin, fluorophores, radiolabels), to a diverse range of type I NKT cell activators (38) (Fig. 4). Through careful design, these modifications do not alter appreciably the functionality of the NKT cell agonist under study and, as a result, these compounds prove to be valuable tools for studying *in vivo* behaviors of the parent antigenic molecules (38, 50). This technology has far-reaching implications, linking other molecules (Bacillus Calmette-Guérin, specific vaccine Ags and toll-like receptor ligands) to NKT cell agonists, and allowing the exploitation of multiple pathways of the immune system concurrently. Of particular relevance is a recently devised mechanism that enables the chemical conjugation of a lipid Ag to CD1d (Fig. 4). Once covalently attached, the Ag cannot dissociate from the CD1d molecule. Consequently, the Ag–CD1d conjugate shows enhanced targeting of NKT cells and reduced off-target effects, in part because immunizing doses can be reduced (51, 52).

**Figure 4:**
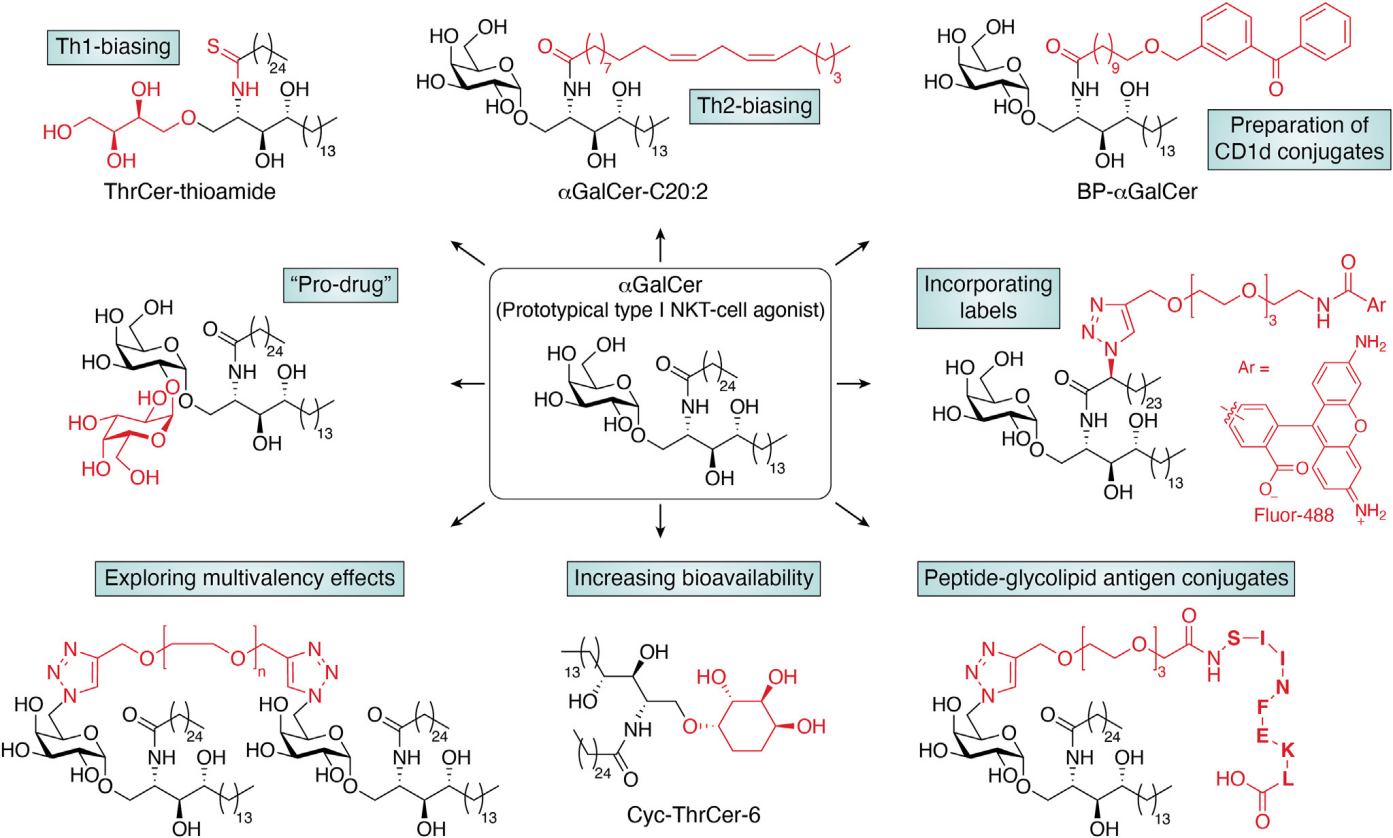
**Strategies for the design of synthetic αGalCer analogs.** Feasible modifications of αGalCer are indicated in *red*. αGalCer can be modified in its headgroup as well as the lipid tails, such as altered chain length, unsaturation, or extra conjugation. αGalCer, α-galactosylceramide.

### TCR recognition of CD1b-lipid

To date, structures of αβTCR–CD1b–lipid complexes follow the corecognition paradigm, as lipid-independent TCR activation is not yet clearly demonstrated. In the four ternary structures published, which include both microbial and self-lipids, the TCRs consistently sit atop of CD1b, with the α- and β-chains perpendicularly interacting with the helices α2 and α1, respectively, creating a “cationic cup” to embrace the polar headgroup of the lipid (Fig. 3*D*) (53, 54, 55, 56). Upon TCR binding, a slight conformational change of CD1b is seen in each case, mostly due to the remodeling of lipid Ag headgroups. There are also differences of TCR-CD1b footprints, whereby the TCR may or may not fully encase the lipid headgroup (56), which reflects the specificity of each TCR toward its cognate lipid. In one example, TCR docking on top of CD1b-self-lipid creates “an escape channel” for the lipid headgroup. This defined mechanism permits TCR cross-reactivity to various self-lipids with different head groups attached to phosphate (54, 55), but it still requires the phosphatidyl group, and so is mechanistically distinct from the fully lipid independent absence of interference mechanism.

### TCR recognition of CD1a-lipid and CD1c-lipid

There is evidence showing that TCR interactions with CD1a and CD1c also require corecognition of CD1 and lipid. The identified *Mycobacterium tuberculosis* lipid Ags including dideoxymycobactin presented by CD1a or PM and MPM presented by CD1c (Fig. 2) are specifically recognized by corresponding T cell lines through TCR contact with the protruding phosphate or mannose unit or both (5, 9, 57, 58). In terms of endogenous lipids, the leukemia-derived methyl-lysophosphatidic acid (Fig. 2) presented by CD1c specifically activates T cells (59, 60). Although the structural data are yet to be obtained, the biochemical function suggests the existence of direct lipid-TCR contact (59, 60, 61, 62).

Notably, TCR recognition of CD1a or CD1c carrying self-lipids can occur without invoking TCR contact with or direct recognition of the lipid. Namely, identification of small headless lipids (63) and the first ternary structure of a TCR–CD1a–lipid complex (64) defined a new structural paradigm, whereby the TCR contacts CD1a without the need to contact the lipid ligand, oleic acid, or lyso-phosphatidylcholine (Fig. 3*E*). Here, the autoreactive TCR binds over the A′ roof of CD1a, thereby “missing” the F′ portal, implying that this mode of direct TCR-CD1a binding would not be impacted by lipids that typically protrude from the F′ portal—these lipids are termed “permissive.” However, certain classes of lipids, including sulfatide and SM, block the T cell response by altering the structure of the A′ roof of CD1a to disrupt the binding of the TCRs or by showing marked protrusion from the F′ portal to block any TCRs moving toward the A′ roof. Thus, SM and sulfatide lipids are termed “nonpermissive” as they predominantly block TCR docking. Direct autoreactivity of TCRs to CD1a binding is thought to be a general property of CD1a-restricted T cells in the skin, where CD1a is abundantly expressed (4, 65). Moreover, the first structure of a γδTCR–CD1a complex also demonstrates a lipid-independent manner of recognition, which occurs due to a highly lateral approach (Fig. 3*F*) (66).

Similarly, a structure of a TCR–CD1c–lipid complex (11) demonstrated the interaction between the αβTCR with CD1c without contacting the lipid ligands, monoacylglycerol, or the spacer lipids (Fig. 3*G*). Here, the autoreactive TCR sat directly above the F′ portal of CD1c, and as such lipids with large or charged headgroups such as PM, MPM, phosphatidylcholine, and SM are nonpermissive for binding while only small lipids that sit snugly inside the CD1c cleft are permissive (11). Collectively, these related studies of CD1c and CD1a defined an “absence of interference” binding mode for autoreactive αβTCRs.

## Conclusions

While there are hundreds of structures of TCR–peptide–MHC complexes, the structural database of TCR–CD1–lipid complexes, with the exception of the NKT TCR-CD1d-lipid structural repertoire, is much less complete. As such, structural models are still evolving. Presently there is only one ternary αβTCR–CD1a and αβTCR–CD1c complex, so the generalities of autoreactive αβTCR-CD1 recognition are unclear. Further how CD1a- and CD1c-restricted foreign lipids are recognized by αβTCRs is unknown. The studies on TCR-CD1b-lipid are slightly more advanced, with a few ternary complexes for a foreign lipid and self-lipids being determined. Despite this major knowledge gap, the fundamental principles of TCR-CD1-lipid recognition can deviate from that of TCR-MHC-peptide. Most saliently, this includes breaking of the TCR corecognition paradigm of MHC-restricted immunity. This lack of corecognition in CD1a-mediated TCR autoreactivity has led to the discovery of natural lipid blockers, namely SMs (4). Subsequently, this has led to the synthetic design of more potent SM blockers that can reduce the autoreactive CD1a-mediated T cell response in skin, suggesting potential promise as therapeutic agents to treat inflammatory skin conditions. Moreover, mAbs directed toward the CD1a A′ roof have shown to be efficacious in reducing inflammation in an *in vivo* mouse model (67). However, a greater understanding of the basic biology of CD1-restricted T cells in humans is required to fully realize its translational potential.

## Conflict of interest

The authors have patents describing biological and small molecule blockers of the TCR-CD1a response.
